# Full Genome Sequencing and Genetic Characterization of Eubenangee Viruses Identify Pata Virus as a Distinct Species within the Genus *Orbivirus*

**DOI:** 10.1371/journal.pone.0031911

**Published:** 2012-03-15

**Authors:** Manjunatha N. Belaganahalli, Sushila Maan, Narender S. Maan, Kyriaki Nomikou, Ian Pritchard, Ross Lunt, Peter D. Kirkland, Houssam Attoui, Joe Brownlie, Peter P. C. Mertens

**Affiliations:** 1 Vector-borne Viral Diseases Programme, Institute for Animal Health, Woking, Surrey, United Kingdom; 2 Australian Animal Health Laboratory, CSIRO, Geelong, Victoria, Australia; 3 Elizabeth Macarthur Agricultural Institute, Camden, New South Wales, Australia; 4 Department of Pathology and Infectious Diseases, Royal Veterinary College, North Mymms, Hatfield, Herts, United Kingdom; The University of Hong Kong, Hong Kong

## Abstract

Eubenangee virus has previously been identified as the cause of Tammar sudden death syndrome (TSDS). Eubenangee virus (EUBV), Tilligery virus (TILV), Pata virus (PATAV) and Ngoupe virus (NGOV) are currently all classified within the *Eubenangee virus* species of the genus *Orbivirus*, family *Reoviridae*. Full genome sequencing confirmed that EUBV and TILV (both of which are from Australia) show high levels of aa sequence identity (>92%) in the conserved polymerase VP1(Pol), sub-core VP3(T2) and outer core VP7(T13) proteins, and are therefore appropriately classified within the same virus species. However, they show much lower amino acid (aa) identity levels in their larger outer-capsid protein VP2 (<53%), consistent with membership of two different serotypes - EUBV-1 and EUBV-2 (respectively). In contrast PATAV showed significantly lower levels of aa sequence identity with either EUBV or TILV (with <71% in VP1(Pol) and VP3(T2), and <57% aa identity in VP7(T13)) consistent with membership of a distinct virus species. A proposal has therefore been sent to the *Reoviridae* Study Group of ICTV to recognise ‘Pata virus’ as a new *Orbivirus* species, with the PATAV isolate as serotype 1 (PATAV-1). Amongst the other orbiviruses, PATAV shows closest relationships to Epizootic Haemorrhagic Disease virus (EHDV), with 80.7%, 72.4% and 66.9% aa identity in VP3(T2), VP1(Pol), and VP7(T13) respectively. Although Ngoupe virus was not available for these studies, like PATAV it was isolated in Central Africa, and therefore seems likely to also belong to the new species, possibly as a distinct ‘type’. The data presented will facilitate diagnostic assay design and the identification of additional isolates of these viruses.

## Introduction

*Eubenangee virus* (EUBV) is one of 22 species (which also represent distinct virus serogroups) of the genus *Orbivirus* within the family *Reoviridae*. The orbiviruses are vector borne, non-enveloped, icosahedral viruses, with genomes composed of 10 segments of linear dsRNA. *Bluetongue virus* (BTV) is the type species of the genus, which also contains 14 currently unclassified strains, some of which may represent additional species [1], [2], [3], [4], [5], [6]. The orbiviruses are primarily transmitted by ticks or haematophagus-insect-vectors (including *Culicoides*, mosquitoes and sand flies) and have a wide host-range that collectively includes domestic and wild ruminants, equines, marsupials, sloths, bats, birds and humans [3], [7], [8].

The *Eubenangee virus* species/serogroup contains viruses that infect both marsupials and cattle, that have also been isolated from mosquitoes and *Culicoides* [7], [9]. Four distinct Eubenangee viruses have been recognized: Eubenangee virus (EUBV); Tilligerry virus (TILV); Pata virus (PATAV); and Ngoupe virus (NGOV) [3]. Eubenangee virus was first isolated in Australia in 1963 from a pool of 31 mosquitoes belonging to 11 species [7]. It was also isolated from *Culicoides spp*. and antibodies to the virus have been detected in cattle and marsupials [7], [10]. Tilligerry virus was isolated in 1971 from *Anopheles annulipes* mosquitoes in Australia [7] and is related to, but distinct from EUBV [11], [12]. The morphology and morphogenesis of Tilligerry virus as examined by electron microscopy appear similar to those described for other orbiviruses [13].

Pata virus was isolated in 1968 from pool of *Aedes palpalis* mosquitoes in the Central African Republic [7]. Ngoupe virus was isolated six years later in 1974 from *Aedes tarsalis* also in the Central African Republic [7]. Unfortunately, Ngoupe virus is not available from the Orbivirus Reference Collection (ORC) at the Institute for Animal Health (IAH) and has not been included in this study.

The disease producing potential of EUBV was not widely explored until the virus was isolated in Australia between October 1998 and March 1999, from wallabies affected by the Tammar sudden death syndrome (TSDS) [9], [14], [15]. The epizootic of TSDS was first noted in captive research colonies near Sydney, then at two zoological gardens in New South Wales and later at a research facility in Queensland [9]. Some of the clinical signs of TSDS resemble peracute bluetongue, however the majority of affected animals died with no premonitory signs and showed accelerated autolysis within 12 hrs of death [15].

Another suspected outbreak of TSDS occurred in western Sydney in 2007 but no virus was isolated [16]. In November 2010, sudden deaths of Tammar wallabies were again reported from several different locations in eastern Australia, with mortality rates as high as 75%, possibly related to the abundance of adult *Culicoides* in the region [17], [18]. Gross post-mortem findings included widespread pulmonary congestion and haemorrhages throughout the body [15], [18]. Eubenangee viruses and/or Wallal viruses were also implicated during 1998, in the sudden onset of subcutaneous edema, pruritis and urticarial lesions of the lower hind legs, tail and ears of captive red kangaroos from the Northern Territory of Australia [9]. Although Eubenangee viruses were identified as etiological agents of TSDS by virus isolation and serological methods during 1998–1999, only partial sequence of VP3 (T2) were available in the database [14], however the full genome of Eubenangee virus has not previously been fully sequenced.

The similar host range, distribution, co-circulation and relatively close serological relationships between EUBV, BTV and EHDV, has occasionally made diagnosis and identification of specific etiological agents difficult by serological methods [19]. However, full genome sequence analyses and phylogenetic comparisons of multiple orbivirus isolates from individual or closely related *Orbivirus* species have supported the development of faster and more reliable virus-species/serogroup/serotype specific diagnostic assays, by conventional and/or real-time RT-PCR [20], [21], [22], [23]. These assays have supported molecular epidemiology studies and in some cases have helped to reassess the taxonomic classification of individual viruses [24], [25].

We report full genome sequence data for Eubenangee virus (EUBV), Pata virus (PATAV) and Tilligerry virus (TILV), which will facilitate development of relevant assays and identification of further isolates that belong to the species *Eubenangee virus*. These data also support the reclassification of PATAV as a distinct new *Orbivirus* species. A proposal to this effect has been sent to the *Reoviridae* Study Group of the International Committee on Taxonomy of Viruses (ICTV).

## Results

### Virus propagation and genomic dsRNA electropherotype

**Figure 1:**
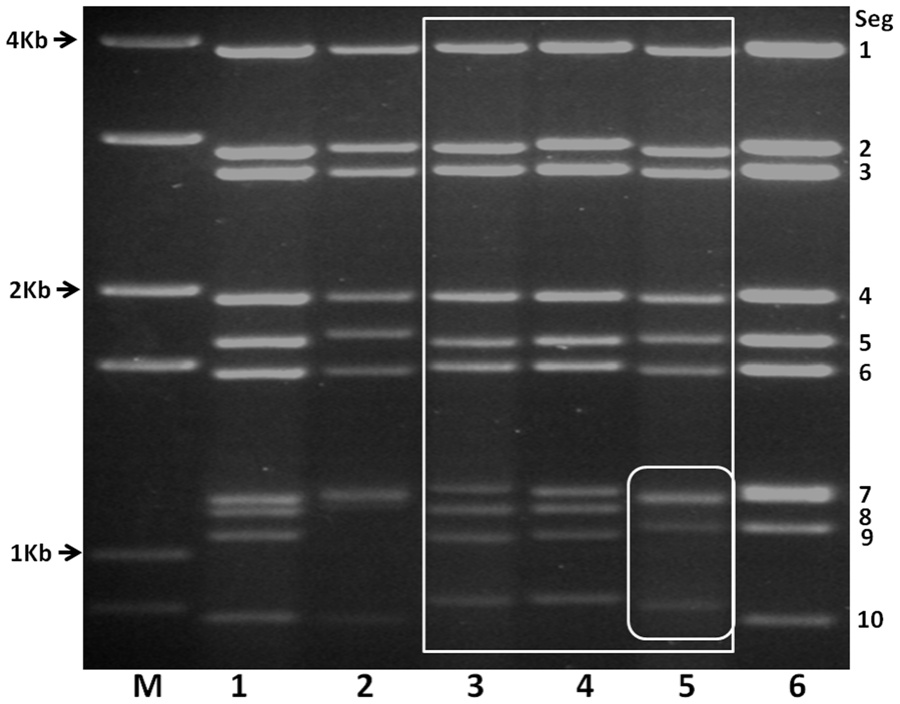
Agarose gel (1%) electrophoretic profile of the dsRNAs of Eubenangee (EUBV), Tilligerry (TILV) and Pata viruses (PATAV) along with those of BTV and EHDV. Lane M = 100 bp Marker; 1 = BTV-1w (LIB2007/07); 2 = EHDV-6w (USA2006/05); 3 = EUBV (AUS1963/01); 4 = TILV (AUS1978/03); 5 = PATAV (CAF1968/01); 6 = EHDV-8e (AUS1982/05).

EUBV, TILV and PATAV isolates (obtained from the Orbivirus Reference Collection (ORC) at IAH (http://www.reoviridae.org/dsRNA_virus_proteins/ReoID/Eubenangee-isolates.htm#EUBV)) induced characteristic cytopathic effects (CPE) in BHK and BSR cell monolayers, between 48–72 hours post infection. Purified dsRNAs of these viruses were analysed by 1% agarose gel electrophoresis (AGE), along with representative BTV and EHDV strains (Figure 1). In this system, EUBV and TILV generated almost identical genome segment migration patterns (Lane 3 and 4 in Figure 1), typical of viruses belonging to the same virus species [3], [26], while PATAV shows little but clear differences in the migration pattern of Seg-7, 8, 9 and 10 (Lane 5 in Figure 1). Seg-7 and 8 of the PATAV co-migrate, while those of EUBV and TILV migrate separately. Although these differences are small, they are comparable in magnitude to the differences between BTV-1w (LIB2007/07 - lane 1), EHDV-6w (USA2006/05 – lane 2), EHDV-8e (AUS1982/05 – lane 6) and EUBV (lane 3).

### Characterisation of the EUBV, TILV and PATAV genome segments

Accession numbers for sequences reported here include: EUBV genome segments JQ070376 to JQ070385; TILV genome segments JQ070366 to JQ070375; PATAV genome segments JQ070386 to JQ070395. The characteristics of the genome segments and encoded proteins are given in Table 1. The G+C content of the full genome of EUBV, TILV and PATAV is 44.99%, 45.143% and 42.2% respectively. The genomes of EUBV and TILV are 19304 bp and 19342 bp respectively, with segments ranging from 3962 to 856 bp, encoding proteins of 1307 to 242 aa length (Table 1). As already suggested (by Figure 1) half of the genome segments (Seg-1, 3, 4, 6 and 8) of EUBV and TILV have identical sizes, while all of the ORFs, except for VP2 and NS4 (Seg-2 and Seg-9), have identical start and stop positions relative to the upstream end, generating proteins of the same length (Table 1).

**Table 1:** Characteristics of dsRNA genome segments and proteins of the Eubenangee (EUBV) (AUS1963/01), Tilligerry (TILV) (AUS1978/03) and Pata (PATAV) (CAF1968/01) viruses.

Seg No.	Seg size (bp)			G+C content (%)			Protein encoded (structure/putative function)	ORFs bp (including stop codon)			No. of amino acids			Predicted protein molecular mass (*kDa*)		
	EUBV	TILV	PATAV	EUBV	TILV	PATAV		EUBV	TILV	PATAV	EUBV	TILV	PATAV	EUBV	TILV	PATAV
**1**	3962	3962	3948	41.77	41.85	40.27	VP1 (Pol)	13–3936	13–3936	12–3923	1307	1307	1303	150.1	150.23	149.59
**2**	2958	2980	2941	42.53	44.13	40.53	VP2	17–2923	17–2944	17–2905	968	975	962	111.76	112.76	111.98
**3**	2773	2773	2770	43.78	42.84	42.06	VP3 (T2)	20–2722	20–2722	18–2717	900	900	899	102.95	103.05	103.28
**4**	1984	1984	1982	43.5	44.46	41.57	VP4 (Cap)	10–1944	10–1944	8–1942	644	644	644	75.11	74.87	75.62
**5**	1763	1770	1785	46.8	46.2	42.35	NS1 (TuP)	30–1700	30–1700	33–1688	556	556	551	65.21	65.15	64.1
**6**	1658	1658	1644	43.37	42.76	42.7	VP5	29–1618	29–1618	29–1612	529	529	527	59.62	59.61	59.28
**7**	1175	1176	1159*	48.26	47.19	43.83	VP7 (T13)	21–1073	21–1073	18–1064	350	350	348	38.26	38.34	38.16
**8**	1128	1128	1177*	47.16	47.16	42.4	NS2 (ViP)	29–1084	29–1084	20–1132	351	351	370	39.12	39.26	43.62
**9**	1047	1050	1083	46.32	48.38	43.03	VP6 (Hel)	17–1003	17–1006	15–1037	328	329	340	35.67	35.67	37.2
							NS4§	114–368	117–383	265–507	84	88	80	9.8	10.36	10
**10**	856	861	851	46.5	46.46	43.24	NS3	20–748	20–748	21–734	242	242	237	26.47	26.5	26.4
							NS3a	95–748	95–748	75–734	217	217	219	23.5	23.6	24.17

* - Variability in the genome segments—Seg-7 of PATAV encodes NS2 and Seg-8 encodes VP7.

§ -NS4 protein, a novel non-structural protein expressed from the Seg-9 of orbiviruses [38].

The overall size of PATAV genome is 19340 bp, with genome segments that range from 3948 bp to 851 bp, encoding proteins of 1303 aa to 237 aa length. All of the PATAV RNAs and ORFs (except for Seg-4) have different sizes and positions from those of EUBV and TILV, leading to the production of proteins with different sizes. The coding assignments of EUBV, TILV and PATAV genome segments are similar to those of BTV and the other *Culicoides* borne orbiviruses, with the exception of PATAV Seg-7 and 8, which have changed their relative order of size, compared to those of TILV and EUBV (Table 1).

The genome segments of all three viruses have shorter 5′ non coding regions (NCR) (9–28 bp in EUBV and TILV; 7–32 bp in PATAV) than 3′ NCRs (29–111 or 29–116 bp in EUBV or TILV; 28–120 bp in PATAV) with conserved terminal hexanucleotides that are comparable to, but in some cases not identical to those of BTV or EHDV (Table 2). Two nucleotides at the 5′ end and three nucleotides at the 3′ end are fully conserved (5′-GU……UAC-3′) while the first and last 2 nucleotides of each genome segment are also inverted complements, characteristics of the genus *Orbivirus* (http://www.reoviridae.org/dsRNA_virus_proteins/CPV-RNA-Termin.htm). These results confirm an early report of the conserved termini of Tilligerry virus genome segments (5′-GU–A………….AC-UAC-3′) as identified by enzymatic digestion and electrophoresis [27].

**Table 2:** Conserved terminal sequences of EUBV, TILV and PATAV genome segments.

Virus species/isolate	Conserved RNA terminal sequences (positive strand)
*Bluetongue virus* *	5′-GUUAAA…………………. ^A^/_G_CUUAC-3′
*Epizootic haemorrhagic disease virus* *	5′-GUUAAA…………………..^A^/_G_CUUAC-3′
Eubenangee virus	5′-GU^U^/_A_^A^/_U_AA…………….. ^A^/_C_C^U^/_A_UAC-3′
Tilligerry virus	5′-GU^U^/_A_^A^/_U_AA……………..AC^U^/_A/C_UAC-3′
Pata virus	5′-GU^U^/_A_AAA………………… ^C^/_A_CUUAC-3′

* From Mertens et al., [3].

Most of the ORF's of the EUBV and TILV genome segments have a strong Kozak sequence (RNN**ATG**G), except for the first ORFs of Seg-9 (CTG**ATG**A) and Seg-10 (G^C^/_A_T**ATG**T), both of which have a ‘weak’ context, but also encode additional smaller proteins from downstream initiation sites (generating NS4 and NS3a respectively). The ORF's of PATAV genome segments have the initiation sequence RNN**ATG**, followed by ‘G’ in Seg-1,5,6,7,8 (strong Kozak context); ‘A’ in Seg-2, 3 (adequate Kozak context); ‘C’ in Seg-4, and -10, and ‘T’ in Seg-9 (weak context).

#### Phylogenetic comparisons of orbivirus subcore-shell ‘T2’ proteins

**Figure 2:**
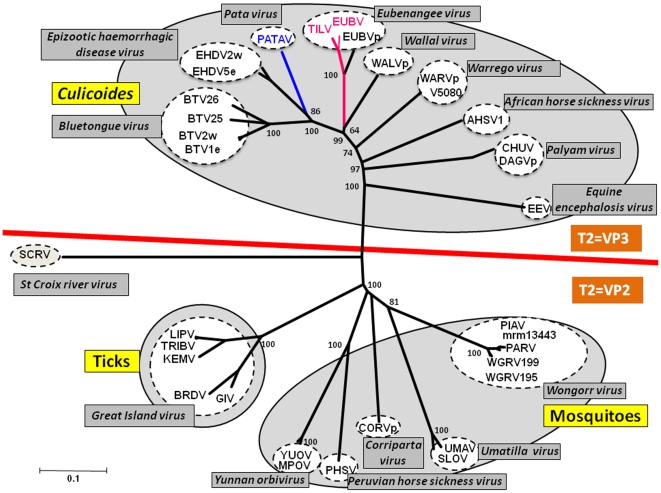
Unrooted Neighbour-joining tree comparing orbivirus T2 protein sequences. The tree was constructed using distance matrices, generated using the p-distance determination algorithm in MEGA 5 (1000 bootstrap replicates) [58]. Since many of the available sequences are incomplete, the analysis is based on partial sequences (aa 393 to 548, numbered with reference to the aa sequence of BTV VP3 (T2)). The numbers at nodes indicate bootstrap confidence values after 1000 replications. The tree shown in Figure 3 was drawn using same parameters. The EUBV and TILV isolates characterised in this study are shown in red font and PATAV is shown in blue font. Full names of virus isolates and accession numbers of T2 protein sequences used for comparative analysis are listed in Table S1 (supplementary data). ‘e’ and ‘w’ after serotype number indicate eastern and western strains, respectively.

In EUBV, TILV and PATAV, VP3 (encoded by Seg-3) was identified as the virion's sub-core-shell ‘T2’ protein, by comparisons with BTV proteins using BlastX. Nucleotide (nt) and amino acid (aa) identities in VP3(T2) of EUBV, TILV and PATAV, as compared to other orbiviruses, are given in Table 3 and 4. Unrooted neighbour-joining (NJ) trees were constructed using p-distance algorithm and pairwise deletion parameters for the T2 protein sequences of the different orbiviruses listed in the Table S1 (supplementary data). Two major clusters were identified (Figure 2): in which VP3(T2) of the *Culicoides* transmitted viruses is encoded by Seg-3 (including BTV, African horse sickness virus [AHSV], Epizootic haemorrhagic disease virus [EHDV], *Wallal virus* [WALV], *Eubenangee virus* [EUBV], *Warrego virus* [WARV] and *Palyam virus* [PALV]); while in the second group, VP3(T2) is encoded by Seg-2 of two orbivirus sub-groups that are transmitted either by ticks (Great island virus [GIV]), or by mosquitoes (Corriparta [CORV], Wongorr virus [WGRV], Peruvian horse sickness virus [PHSV], Yunnan orbivirus [YUOV]). *St Croix River virus* [SCRV] branches separately, representing a more distantly related orbivirus that may be a tick-virus rather than a tick-borne-virus [28]. EUBV and TILV viruses cluster together and are distinct from the other *Orbivirus* species in the ‘*Culicoides* borne group’, although they cluster with other species that infect marsupials (Wallal virus and Warrego virus) (Figure 2).

**Table 3:** Percent nucleotide (nt) and amino acid (aa) identities between Eubenangee, Tilligerry, Pata, Bluetongue and Epizootic Haemorrhagic disease viruses.

Seg No.	Protein	EUBV vs TILV		EUBV vs BTV		EUBV vs EHDV		PATA vs TILV		PATA vs BTV		PATA vs EHDV		EUBV vs PATA	
		nt	aa	nt	aa	nt	aa	nt	aa	nt	aa	nt	aa	nt	aa
**1**	VP1(Pol)	78.24	92.1	61.1–61.65	63.5	61.7–62.57	64.3–63.3	62.9	64.7	64.3–64.5	68.7–70.3	65.51–66.5	72.4	62.86	65
**2**	VP2	57.17	52.22	35.6–36.52	16.4–17.8	34.75–35.7	16.7–17.5	36.37	17.9	40–41	24.7–26.1	37.5–37.9	19.6–20.5	38.03	18.34
**3**	VP3 (T2)	80.66	95.66	63.36–64.5	68.3–69	65.094–66	69.3–69.8	65.02	70.63	67.5–69.3	74.97–76.86	70.9–71.4	80.2–80.7	64.79	70.16
**4**	VP4	79.28	90.84	56–57.3	54.5–56.2	56.6–57.45	55.1–55.6	56.36	55.9	60.2–61.2	60.8–63.2	60–60.1	62.1–64.3	57.37	55.59
**5**	NS1	73.8	80.04	43.08–44.02	27.5–28.4	43.2–44.3	26.7–27.7	43.24	28.36	54.19–56.42	47.27–49.8	55.4–55.9	49–50	42.99	29.45
**6**	VP5	76.48	88.1	55.3–56.7	51.4–52.8	57.3–57.7	52.7–53.3	59.28	55.51	60–61.82	58.7–61.1	60–61.6	61.7–61.9	57.33	55.32
**7**	VP7(T13)	82.21	95.14	55.6–56.96	51.6–53.58	55.1	53.9–54.4	57.94	57.18	61.9–64.3	64.3–64.94	63.9–65.03	66.9–67.5	57.65	57.47
**8**	NS2	77.66	82.05	41.04–51.57	38–40.37	47.07–50.18	37.6–39.5	49.62	40.06	55.1–56.92	49.7–51.5	56.6–57.2	51.1–52	50.85	40.67
**9**	VP6	75.36	70.12	47.3–49	32.6–35	46.5–48.85	33.7–35	50.8	33.11	53–56.5	40–41.9	54–55.56	40.6–42.7	48.66	32.11
**10**	NS3	67.87	68.6	52.2–52.8	41.5–43.7	49.1–52.1	37.9–38.8	52.7	47.44	62.2–62.8	56.3–58-5	58.9–59.8	53.8–55.56	55.1	44.87

**Table 4:** Inner core protein (T2) amino acid (aa) identities (%) between EUBV, TILV and PATAV with other orbiviruses.

Virus/nt accession no	EUBV(AUS1963/01)	TILV(AUS1978/03)	PATAV(CAF1968/01)	Vectors
EUBV/AUS1963/01	100.00	95.66	70.16	
TILV/AUS1978/03	95.66	100.00	70.63	
PATV/CAF1968/01	70.16	70.63	100.00	
BTV1e/DQ186822	68.85	69.00	76.42	
BTV2w/DQ186826	69.08	69.22	76.86	
TOV/GQ982523	68.30	68.44	75.31	
BTV26/HM590643	68.41	68.89	74.97	
EHDV2w/AM744999	69.38	69.86	80.20	
EHDV5e/AM745029	69.82	70.86	80.76	*Culicoides*
EUBVp/AF530087	89.78	90.87	71.74	
WALVp/AF530084	77.58	76.87	74.02	
WARVp/AF530083	70.04	70.45	69.64	
V5080/EF213555	69.80	70.61	69.80	
AHSV1/AM883166	62.29	61.89	59.07	
EEV/FJ183386	52.17	52.11	50.39	
CHUV/NC_005989	58.95	58.44	57.51	
DAGVp/AF530085	67.14	67.14	63.81	
YUOV/NC_007657	37.79	37.31	36.90	
PHSV/NC_007749	36.30	36.26	36.41	
WGRV195/U56990	42.23	41.75	38.35	
WGRV199/U56991	42.33	41.86	37.67	
MRM13443/U56992	39.76	39.76	37.80	
PARV/U56993	40.67	40.19	38.76	*Mosquitoes*
PIAV/U56994	40.59	40.10	38.61	
MPOV/EF591620	37.57	37.08	36.45	
CORVp/AF530086	41.77	41.77	43.90	
UMAV/HQ842620	36.01	36.08	34.34	
SLOV/EU718677	35.67	35.75	34.23	
BRDV/M87875	35.86	35.71	34.74	
GIV/HM543466	37.64	37.71	36.30	
KEMV/HM543482	36.19	35.71	35.86	*Ticks*
LIPV/HM543476	35.97	35.48	36.08	
TRBV/HM543479	36.08	35.60	35.86	
SCRV/AF133432	22.17	22.37	22.37	Probably Ticks

In contrast VP3(T2) of PATAV branches independently from EUBV and TILV within the *Culicoides* borne group, clustering more closely with EHDV and BTV. Although EUBV, TILV and PATAV were initially isolated from mosquitoes, EUBV has also been isolated from *Culicoides* [7] and all three viruses group more closely with the *Culicoides* borne orbiviruses, suggesting that these insects may act as their biological vectors.

#### Phylogenetic comparisons of VP1(Pol) proteins

**Figure 3:**
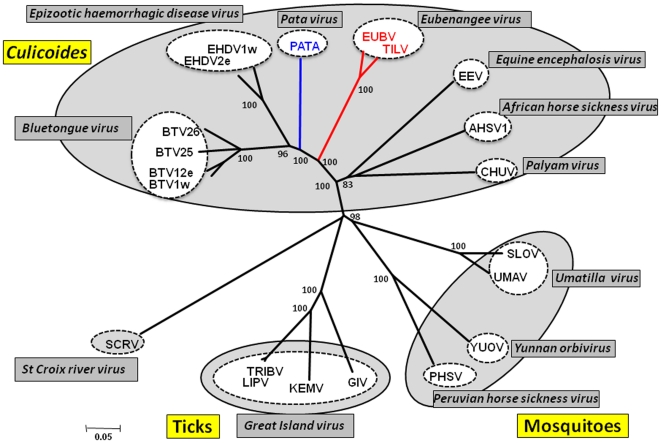
Neighbour-joining tree showing the relationships between amino acid sequences of VP1 polymerase (RdRp) protein of different orbiviruses. The EUBV and TILV isolates characterised in this study are shown in red font, and PATAV is shown in blue font. Full names of virus isolates and accession numbers of polymerase sequences used for comparative analysis are listed in Table S2 (supplementary data). ‘e’ and ‘w’ after serotype number indicate eastern and western strains, respectively.

The viral RNA dependent RNA polymerase (Pol) gene encoded by Seg-1 of all orbiviruses is highly conserved within the genus. Phylogenetic comparisons of this genome segment and protein have been used to classify new virus isolates at the species and genus level [1], [28], [29]. The aa sequence of EUBV, TILV and PATAV VP1(Pol) and homologous proteins from representatives of the other *Orbivirus* species were compared and used to construct an unrooted NJ phylogenetic tree (Figure 3) (the accession numbers used are listed in Supplementary Table S1; percentage identities are given in Table S2 - supplementary data). As already observed for VP3(T2) (Figure 1), VP1(Pol) of EUBV, TILV and PATAV again group with other *Culicoides* borne orbiviruses. EUBV and TILV cluster very closely together (consistent with membership of a single virus species), while PATAV branches separately (Figure 3).

#### Phylogenetic comparisons of outer-core VP7(T13) proteins

The core surface protein VP7(T13) of BTV is a strongly immunodominant antigen and is the primary determinant of virus-serogroup [30]. The aa sequence of this protein, which is highly conserved within each *Orbivirus* species, has provided a target for development of serogroup-specific serological diagnostic assays [30]. The aa sequence of VP7(T13) from EUBV, TILV and PATAV, were compared to those of other orbiviruses (accession numbers are given in Table S1 - supplementary data). An unrooted phylogenetic tree, constructed using the T13 aa sequences (data not shown), shows the same topology as trees constructed for the T2 and VP1(Pol) proteins, showing separate branching of PATAV from EUBV and TILV and grouping all three with the *Culicoides* borne viruses.

The VP7(T13) protein of EUBV, TILV and PATAV shares 37.93% to 67.53% aa sequence identity with the *Culicoides* borne orbiviruses; 20.63% to 26.01% aa identity with mosquito borne orbiviruses; 21.5% to 24.5% aa identity with the tick borne orbiviruses; and only 20.41%–22.65% aa identity with the most distantly related SCRV (Table S3-supplementary data).

#### Relationships between EUBV, TILV and PATAV

Sequence identity levels were calculated for RNAs and proteins of EUBV, TILV and PATAV, in comparison to members of other *Orbivirus* species, using the p-distance and pairwise deletion parameters in MEGA5 (Table 3 and 4). Four of the EUBV and TILV proteins share >90% aa identity: with 95.66% in the subcore-shell ‘T2’ protein; 95.14% in the core-surface and serogroup specific antigen VP7(T13); 92.1% in the polymerase VP1(Pol); and 90.84% in the capping enzyme and transmethylase VP4(CaP) (Tables 3 and 4). Based on these data, previous comparisons of other *Orbivirus* species confirm that EUBV and TILV belong to the same virus species (Figure 1, 2 and 3, Table 4). In contrast the sequence of outer-capsid protein VP2 is highly variable between EUBV and TILV, with only 52.2% aa identity. Based on data from BTV [25], [31], [32] this indicates that they represent distinct virus ‘types’ within the same species. The second outer capsid protein, VP5 (encoded by Seg-6) is also the second most variable protein of BTV, although it can sometimes show high levels of identity between distinct serotypes [31]. Comparisons of TILV and EUBV showed that within this limited sample group, VP5 was less variable (88.1% aa identity) than either NS3 or VP6(Hel), which have 68.6% or 70.12% aa identity respectively (Table 3).

PATAV shares <70.2% identity in VP3(T2); <65% identity in the polymerase VP1(Pol) and <57.5% aa identity in VP7(T13) with TILV or EUBV (Table 3 and 4), consistent with membership of a different *Orbivirus* species/serogroups [1], [31], [33]. Among the other orbiviruses, Pata virus is most closely related to EHDV and BTV with 66.9% to 80.7% and 64.3% to 76.86% aa identity respectively in these conserved proteins (Table 3 and 4), while EUBV and TILV share between 51.6% to 69.8% aa identity with BTV and EHDV (Table 3), levels that are also consistent with membership of distinct *Orbivirus* species.

Most of the genome segments in EUBV and TILV are monocistronic encoding single proteins from single open reading frames (ORF). However, Seg-10 of both viruses has two in-frame initiation sites at, or near the start of the same ORF leading to translation of NS3 and NS3a (Table 1). A similar coding strategy has previously been reported for Seg-10 of BTV which encodes two related non-structural proteins (NS3 and NS3a) that are more abundantly expressed in insect rather than in mammalian cells [34], [35], [36]. Recently, a novel non-structural protein ‘NS4’ of BTV and several other orbiviruses (which varies between 10 kDa to 22.5 kDa), was identified encoded by an alternative downstream ORF (from +2 frame) on Seg-9 (which also encodes VP6-Helicase) [33], [37], [38].

Seg-9 of EUBV, TILV and PATAV also has an additional down-stream ORF (in reading-frame +2) (Table 1). The 2nd ORF on Seg-9 of EUBV and TILV are in approximately the same position (at 114–368 bp (84 aa) and 117–383 bp (88 aa) respectively) and contain moderately strong Kozak sequences in each case (GGA**ATG**A and GAG**ATG**A). In contrast the 2nd ORF of PATAV is much further downstream on Seg-9, at 265–504 bp (80 aa), and has a weak Kozak context (AAG**ATG**C), similar to those observed for NS4 of BTV and some other orbiviruses [38].

Hydrophobicity profiles of NS4 proteins from BTV, EHDV, GIV, EUBV, TILV and PATAV show broadly similar patterns of conserved domains indicating that they are generally hydrophilic (Figure S1-Supplementary data). Sequence analyses indicate that PATAV NS4 is structured as coiled-coil (cc) domains over its entire length, while EUBV NS4 contains two cc domains between aa 1 to 14 and 35 to 84 and TILV NS4 contains two cc domains between aa 2 to 17 and 33 to 88. A Pfam search indicates similarities between NS4 of PATAV, with cc domains of PROX1, a protein that interacts with EP300, which in turn interacts with nuclear components [39]. These similarities suggest that NS4 of PATAV may also interact with the nucleus. Like NS4 of the other orbiviruses [38], potential monopartite and bipartite nuclear localisation signals (NLS) were identified in NS4 of EUBV, TILV and PATAV (Table S4-supplementary data). A Pfam search also identified matches between NS4 of EUBV and the integrase of bacteriophage lambda, indicating possible interaction between NS4 and nucleic acids. It has been experimentally shown that NS4 of BTV and GIV both bind DNA [38].

## Discussion

Parameters recognised by the International Committee on Taxonomy of Viruses (ICTV) for the ‘polythetic definition’ of individual *Orbivirus* species include: the reassortment of genome segments; genome segment migration patterns during 1% agarose gel electrophoresis (AGE); conserved terminal nucleotide sequences; serological cross-reactions; comparison of homologous genome segments or proteins by sequence analysis or cross-hybridisation; host and vector range and the nature of clinical signs induced [3]. The majority of the existing *Orbivirus* species were initially recognised as distinct ‘serogroups’. However, reference antisera for these existing species are not widely available, making serological identification of new virus isolates more difficult. Data concerning the potential for reassortment of new orbivirus isolates with members of existing species would also require access to reference strains and would be very labour intensive. In contrast, nt and deduced aa sequence data generated for each species can be easily and reliably disseminated and can be used to analyse and identify new or existing virus isolates [1], [25], [28], [29], [40].

Viruses within a single *Orbivirus* species/serogroup usually have similar dsRNA migration profiles during in 1% agarose gel electrophoresis (AGE) [3]. EUBV, TILV and PATAV show a 3-3-3-1 RNA migration during AGE, broadly similar to those of BTV and EHDV, suggesting relatively close relationships with the members of these virus species. The RNA-AGE migration patterns of EUBV and TILV are almost identical, suggesting that they belong to the same species. However, PATAV shows a number of differences, particularly in the migration of Seg-7 to 10, compared to EUBV or TILV, suggesting that it belongs to a different species.

The rationale for the current inclusion of Pata virus (PATAV) in the species *Eubenangee virus* does not appear to be recorded. It does not cross-react strongly in serological assays with other EUBV isolates and it is not closely related to EUBV by hybridrization [19], [41], [42], leading to suggestions that the current classification of PATAV should be re-examined [19], [42], [43]. PATAV does show low level cross-reactivity in complement fixation (CF) tests with BTV and EHDV serogroups [19], [43] and it has been suggested that it could be reclassified as an EHDV [19], [43]. However, no cross hybridization or reassortment was detected between PATAV and BTV or EHDV [43].

Low level cross-reactions have previously been shown between EUBV, and BTVs or EHDVs in cross-hybridization and serological assays (including CF and agar gel immunoprecipitation tests) [19], [43], [44], reflecting a common ancestry [12], [42], [45], [46], [47]. PATAV is not closely related to other Eubenangee viruses by hybridization [42], and hybridization or gene reassortment data indicate that it is not a BTV or EHDV [43].

Recent advancements in molecular biology and sequencing technologies [26] have allowed phylogenetic comparisons to be used as major tools for orbivirus identification, development of diagnostic tests and taxonomic classification. Sequence data generated for conserved orbivirus genes (e.g. the T2, Polymerase or T13 protein genes) have previously been used for phylogenetic comparisons and taxonomic classification [1], [40]. However, a lack of sequence data for reference strains of all 22 *Orbivirus* species remains a barrier for molecular identification, diagnostic assay development and taxonomic classification of novel isolates.

This study was therefore designed to generate full-genome sequences for available members of the species *Eubenangee virus* (EUBV, TILV and PATAV) as part of a wider programme generating representative sequences for all of the different *Orbivirus* species [1], [24], [28], [29], [31], [33], [40], [48], [49], [50].

Although EUBV and TILV have been isolated from both *Culex annulirostris* and *Culicoides marksi,* EUBV multiplied to high titres in the recognised orbivirus vector *Culicoides variipennis* after both intra-thoracic inoculation and oral ingestion, but only replicated in *C. nubeculosus* (usually regarded as a non-vector species) after intra-thoracic inoculation [51]. Earlier studies have reported a highly conserved Arg-Gly-Asp (RGD) motif at position 168–170 in the VP7(T13) core-surface protein of the *Culicoides* borne orbiviruses BTV, EHDV and AHSV, suggesting that it could have a role in core particle attachment to *Culicoides* cells [48], [52], [53]. An RGD motif was not observed in EUBV and TILV, but was present at position 168–170 in VP7(T13) of PATAV, reflecting its closer overall similarity to BTV and EHDV, and potentially implicating *Culicoides spp.* as biological vectors.

The G+C content of the mosquito borne orbiviruses is between 36.72% in Peruvian horse sickness virus (PHSV) and 41.55% in Yunnan orbivirus (YUOV), in the tick borne orbiviruses it is between 57.29% in Great Island virus (GIV) and 51.93% in St Croix River virus (SCRV), while the *Culicoides* borne orbiviruses have intermediate G+C content from 39.89% in Chuzan virus (CHUV) to 45.89% in equine encephalosis virus (EEV) [1]. The G+C content of EUBV, TILV and PATAV is 44.99%, 45.143% and 42.2% respectively, within the range of the *Culicoides* borne orbiviruses, again suggesting *Culicoides* as a biological vector.

The orbivirus sub-core-shell ‘T2’, polymerase ‘Pol’ and outer-core ‘T13’ proteins are highly conserved with >83%, >73% and >73% aa identity respectively within a single *Orbivirus* species [1], [28], [31]. The highest inter-species identity levels for T2 and Pol that have so far been detected (80% and 73% in T2 and Pol respectively) are between isolates of BTV and EHDV, reflecting their close relationship, as well as the large number of different isolates that have been sequenced from these two virus species [1], [3], [33]. EUBV and TILV share >92% aa identity in these highly conserved proteins, confirming that they belong to the same virus species. In contrast they show lower levels of aa identity with members of other *Orbivirus* species, with highest similarities to EHDV and BTV. PATAV shows lower levels of identity with the Eubenangee viruses (EUBV and TILV) supporting earlier hybridization and reassortment studies [42], [43] indicating that PATAV is a not a member of this virus species. Amongst the other orbiviruses, PATAV also shares highest aa identity levels with EHDV and BTV proteins (Table 3) indicating that it belongs to a closely related but distinct species.

The orbivirus NS4 protein is highly variable in size, ranging from 77 aa in BTV to 190 aa in GIV [38]. NS4 of EUBV, TILV and PATAV is relatively small size at 84 aa, 88 aa and 80 aa respectively. Further *in vivo* studies will confirm the functional role of these proteins in the infected cell.

Previous phylogenetic studies of mitochondrial genes have indicated that ticks represent ancestors for other arthropods [54]. Phylogenetic analysis of the conserved genes and proteins (Figure 2 and 3), shows phylogenetic segregation between the orbiviruses that are transmitted by *Culicoides* (which have VP3(T2)) and those transmitted by ticks or mosquitoes, (in which the homologous protein is VP2(T2)). This difference appears to be primarily due to the presence of a significantly larger outer-capsid ‘VP2’ protein in the *Culicoides* borne orbiviruses. Analyses of the orbivirus T2 (Figure 2), polymerase proteins (Figure 3) and T13 proteins (data not shown), show similar phylogenetic separation of the *Culicoides,* mosquito and tick borne orbiviruses, providing evidence of co-evolution or ‘co-speciation’ with their vectors [33].

Data presented here confirm that EUBV and TILV are both members of the *Eubenangee virus* species/serogroup. However, they contain <52% aa identity in outer capsid protein VP2. Based on data from BTV and EHDV [49], [50], this indicates that they belong to different serotypes - EUBV-1 and EUBV-2 respectively. These data also support the reclassification of Pata virus as the prototype (PATAV-1) of a distinct and novel *Orbivirus* species. A proposal to recognise the species ‘Pata virus’ has therefore been sent to the Reoviridae study Group of ICTV. The availability of full-genome sequences for these reference strains, will help to identify novel Eubenangee and Pata viruses, providing a basis for molecular epidemiology studies to determine strain movements and help identify relevant arthropod vectors. They will also help in the development of improved diagnostic assays for the TSDS (e.g. by RT-PCR).

## Materials and Methods

### Virus propagation

Isolates of Eubenangee viruses which had been stored at −80°C, were obtained from the Orbivirus Reference Collection (ORC) at the Institute for Animal Health (IAH), including: EUBV (ORC Strain Number: AUS1963/01); TILV (AUS1978/03); and PATAV (CAF1968/01). These samples were taken from naturally infected animals in the field, by qualified veterinarians, as part of normal diagnostic testing procedures in the respective countries and did not therefore require Ethics Committee approval. TILV was propagated in BHK-21 cells (clone 13 obtained from European Collection of Animal cell Cultures (ECACC – 84100501), while EUBV and PATAV were grown in BSR cells (a clone of BHK) [55], at 37°C in Dulbecco's minimum essential medium (DMEM) supplemented with antibiotics (100 units/ml penicillin and 100 µg/ml streptomycin) and 2 mM glutamine. Infected cell cultures were incubated at 37°C until they show widespread (100%) cytopathic effects (CPE). Then viruses were harvested, aliquoted and used for the viral dsRNA extraction, or stored in the orbivirus reference collection (ORC) at −80°C.

### Preparation of viral dsRNA

Intact genomic dsRNA was extracted from EUBV, TILV or PATAV infected cell cultures, using a guanidinium isothiocyanate extraction procedure, as described by Attoui et al [56]. Briefly, the infected cell pellet was lysed in 1 ml of commercially available TRIZOL® reagent (Invitrogen), 0.2 volume of choloroform was added, mixed by vortexing and the mixture incubated on ice for 10 min. The supernatant, containing total RNA, was separated from the cellular debris and DNA by centrifuging at 10,000× *g* for 10 min at 4°C. Single stranded RNA (ssRNA) was removed by 2 M LiCl precipitation at 4°C overnight, followed by centrifugation at 10,000× g for 5 min. An equal volume of isopropanol, containing 750 mM ammonium acetate, was added to the supernatant then mixed and the viral dsRNA allowed to precipitate for a minimum of 2 hours at −20°C. The dsRNA was pelleted by centrifugation at 10,000× *g* for 10 min. The pellet was washed with 70% ethanol, air dried and suspended in nuclease free water (NFW). The RNA was either used immediately or stored at −20°C.

### Reverse transcription of dsRNA, and PCR amplification

Viral genome segments of EUBV, TILV and PATAV were reverse-transcribed into cDNA using the full-length amplification of cDNAs (FLAC) technique described by Maan et al [26]. Briefly, a 35 base oligonucleotide ‘anchor-primer’, with a phosphorylated 5′ terminus, was ligated to the 3′ ends of the viral dsRNAs using the T4 RNA ligase, followed by reverse transcription using RT system (Promega). The resulting cDNAs were amplified using complementary primers to the anchor primer. The resulting cDNA amplicons were analyzed by agarose gel electrophoresis. For cloning purposes, a high fidelity KOD polymerase enzyme (Novagen) was used in the PCR.

### Cloning and sequencing of cDNA segments

Amplified cDNAs were purified and cloned. TILV genes were cloned into the Strataclone blunt-end PCR cloning vector ‘pSC-B-amp/kan’ supplied in the StrataClone Blunt PCR cloning kit (Stratagene). EUBV and PATAV amplicons were cloned into the ‘pCR®-Blunt’ vector supplied with the Zero Blunt® PCR Cloning Kit (Invitrogen). Recombinant plasmid-vectors containing TILV inserts were transformed into Solopack® competent cells (Agilent Technologies), while EUBV and PATAV plasmids were transformed into One Shot® TOP10 competent cells, supplied with the respective cloning kits. Clones containing relevant inserts were identified by touch PCR using M13 universal primers. Plasmids were extracted from the clones identified, using the QIAprep Spin MiniPrep Kit (Qiagen). The plasmids and PCR products were sequenced using an automated ABI 3730 DNA sequencer (Applied Biosystems).

### Sequence analysis and phylogenetic tree construction

‘Raw’ ABI sequence data, was assembled into ‘contigs’ using the SeqManII sequence analysis package (DNAstar version 5). The ORFs of EUBV and TILV genome segments were identified using EditSeq and NCBI ORF finder, and translated to aa sequences for further analysis. Putative functions were assigned to individual proteins by comparisons to sequences in GenBank and identification of homologous BTV proteins, using BlastX (http://blast.ncbi.nlm.nih.gov/Blast.cgi?CMD=Web&PAGE_TYPE=BlastHome). Multiple alignments of consensus sequences were performed using Clustal X (Version 2.0) [57], Clustal Omega (http://www.ebi.ac.uk/Tools/msa/clustalo/) and MAFFT (http://mafft.cbrc.jp/alignment/server/) to ensure proper alignment. Pairwise distance (aa and nt) calculations and phylogenetic trees constructions were done using MEGA 5 software [58] with the p-distance parameter and neighbor-joining method [59]. The hydrophobicity profile of different NS4 proteins was analysed using the Kyte and Doolittle hydrophobicity plot with a window size of 11 amino acids (aa) [60]. Sequence relatedness to proteins and domains were assessed by comparing with the pfam database (Available at http://pfam.sanger.ac.uk/search). The presence of ‘coiled-coils’ was analysed using the program ‘COILS’ (available at http://www.ch.embnet.org/software/COILS_form.html). Nuclear localisation signals were predicted using cNLS Mapper (available at http://nls-mapper.iab.keio.ac.jp/cgi-bin/NLS_Mapper_form.cgi).

## Supporting Information

Figure S1 **Hydrophobicity profiles of orbivirus NS4 proteins.** Superimposed hydrophobicity profiles based on multiple alignment of orbivirus NS4 amino acid sequences, generated using Clustal X2. The residue numbers are relative to NS4 of GIV. GIV NS4 (red line), BTV NS4 (blue line), EUBV NS4 (green line), TILV NS4 (Magenta line), PATAV NS4 (purple line) and EHDV (black line).
(DOCX)

Table S1 Nucleotide accession numbers for sequences used in phylogenetic analysis.
(DOCX)

Table S2 Percent aa identity of EUBV, TILV and PATAV Polymerase (Pol) protein with other orbiviruses.
(DOCX)

Table S3 Identity levels in the outer-core protein VP7(T13) of EUBV, TILV and PATAV compared to other Orbiviruses.
(DOCX)

Table S4 Nuclear localization signals (NLS) in NS4 of Eubenangee, Tilligerry and Pata viruses.
(DOCX)
