# Supplementary material for: Full Genome Sequencing and Genetic Characterization of Eubenangee Viruses Identify Pata Virus as a Distinct Species within the Genus Orbivirus
Source: PLoS One. 2012 Mar 15;7(3):e31911. doi: 10.1371/journal.pone.0031911 (PMC3305294; doi:10.1371/journal.pone.0031911)
Supplement: Figure S1 — Hydrophobicity profiles of orbivirus NS4 proteins. Superimposed hydrophobicity profiles based on multiple alignment of orbivirus NS4 amino acid sequences, generated using Clustal X2. The residue numbers are relative to NS4 of GIV. GIV NS4 (red line), BTV NS4 (blue line), EUBV NS4 (green line), TILV NS4 (Magenta line), PATAV NS4 (purple line) and EHDV (black line). (DOCX) [file pone.0031911.s001.docx]

**Supplementary data**

171

152

133

114

95

76

57

38

19

-3.0

-2.0

-1.0

2.0

1.0

0.0

Mean hydrophobicity

Residue Number

**Figure S1. Hydrophobicity profiles of orbivirus NS4 proteins.**

Superimposed hydrophobicity profiles based on multiple alignment of orbivirus NS4 amino acid sequences, generated using Clustal X2. The residue numbers are relative to NS4 of GIV. GIV NS4 (red line), BTV NS4 (blue line), EUBV NS4 (green line), TILV NS4 (Magenta line), PATAV NS4 (purple line) and EHDV (black line).
