# Supplementary material for: Full Genome Sequencing and Genetic Characterization of Eubenangee Viruses Identify Pata Virus as a Distinct Species within the Genus Orbivirus
Source: PLoS One. 2012 Mar 15;7(3):e31911. doi: 10.1371/journal.pone.0031911 (PMC3305294; doi:10.1371/journal.pone.0031911)
Supplement: Table S1 — Nucleotide accession numbers for sequences used in phylogenetic analysis. (DOCX) [file pone.0031911.s002.docx]

**Supplementary data**

**Table S1**: Nucleotide accession numbers for sequences used in phylogenetic analysis

| **Species** | **Serotype** | **Isolate or strain** | **Abbreviation** | **GenBank nucleotide accession no.** | **Segment (Protein)** |
| --- | --- | --- | --- | --- | --- |
| ***African Horse sickness virus (AHSV)*** | Serotype-1 | HS29/62 | AHSV1 | FJ183364 | Seg-1 (RdRp) |
|  | Serotype-1 |  | AHSV1 | AM883166 | Seg-3 (T2) |
|  | Serotype-9 | E00605 | AHSV | HM035361 | T13 |
| ***Bluetongue*** *viru****s (BTV)*** | Serotype-6 | USA2006/01 | BTV-6w | GQ506536 | Seg-1 (RdRp) |
|  | Serotype-12 | BTV12/PT/2003 | BTV-12e | GU390658 | Seg-1 (RdRp) |
|  | Serotype-25 | TOV | TOV | GQ982522 | Seg-1 (RdRp) |
|  | Serotype-26 | KUW2010/02 | BTV-26 | JN255156 | Seg-1 (RdRp) |
|  | Serotype-1 | GRE2001/05 | BTV-1e | DQ186822 | Seg-3 (T2) |
|  | Serotype-2 | TUN2000/01 | BTV-2w | DQ186826 | Seg-3 (T2) |
|  | Serotype-25 | TOV | TOV | GQ982523 | Seg-3 (T2) |
|  | Serotype-26 | KUW2010/02 | BTV-26 | HM590643 | Seg-3 (T2) |
|  | Serotype-15 |  | BTV-15e | L11723 | Seg-7 (T13) |
|  | Serotype-6 | USA2006/01 | BTV-6w | GQ506542 | Seg-7 (T13) |
|  | Serotype-25 | TOV | TOV | EU839843 | Seg-7 (T13) |
|  | Serotype-26 | KUW2010/02 | BTV-26 | HM590644 | Seg-7 (T13) |
| ***Corriparta virus (CORV)*** | Corriparta virus | MRM1 | CORV | AF530086**^*^** | Seg-3 (T2) |
| ***Epizootic Haemorrhagic disease virus (EHDV)*** | Serotype-1 | USA1955/01 | EHDV-1w | AM744977 | Seg-1 (RdRp) |
|  | Serotype-2 | AUS1979/05 | EHDV-2e | AM744987 | Seg-1 (RdRp) |
|  | Serotype-2 | CAN1962/01 | EHDV-2w | AM744999 | Seg-3 (T2) |
|  | Serotype-5 | AUS1977/01 | EHDV-5e | AM745029 | Seg-3 (T2) |
|  | Serotype-2 | CAN1962/01 | EHDV-2w | AM745003 | Seg-7 (T13) |
|  | Serotype-2 | AUS1979/01 | EHDV-2e | AM744993 | Seg-7 (T13) |
| ***Equine encephalosis virus(EEV)*** | Bryanston | HS103/06 | EEV | FJ183384 | Seg-1 (RdRp) |
|  | Bryanston | HS103/06 | EEV | FJ183386 | Seg-3 (T2) |
|  | Bryanston | HS103/06 | EEV | FJ183391 | Seg-7 (T13) |
| ***Eubenangee virus (EUBV)*** | Eubenangee virus | In 1074 | EUBV | AF530087**^*^** | Seg-3 (T2) |
| ***Great Island virus(GIV)*** | Great Island virus | CanAr 42 | GIV | HM543465 | Seg-1 (RdRp) |
|  | Great Island virus | CanAr 42 | GIV | HM543466 | Seg-2 (T2) |
|  | Great Island virus | CanAr 42 | GIV | HM543471 | Seg-7 (T13) |
|  | Broadhaven virus |  | BRDV | M87875 | Seg-2 (T2) |
|  | Broadhaven virus |  | BRDV | M87876 | Seg-7 (T13) |
|  | Kemorovo virus | EgAn 1169-61 | KEMV | HM543481 | Seg-1 (RdRp) |
|  | Kemorovo virus | EgAn 1169-61 | KEMV | HM543482 | Seg-2 (T2) |
|  | Lipovnik | CzArLip 91 | LIPV | HM543475 | Seg-1 (RdRp) |
|  | Lipovnik virus | CzArLip 91 | LIPV | HM543476 | Seg-2 (T2) |
|  | Tribec virus |  | TRBV | HM543478 | Seg-1 (RdRp) |
|  | Tribec virus |  | TRBV | HM543479 | Seg-2 (T2) |
| ***Palyam virus (PALV)*** | Chuzan virus |  | CHUV | NC_005990 | Seg-1 (RdRp) |
|  | Chuzan virus |  | CHUV | NC_005989 | Seg-3 (T2) |
|  | D'Aguilar virus | D'Aguilar B8112 | DAGV | AF530085**^*^** | Seg-3 (T2) |
|  | Chuzan virus |  | CHUV | NC_005988 | Seg-7 (T13) |
| ***Peruvian Horse sickness virus (PHSV)*** | PHSV |  | PHSV | DQ248057 | Seg-1 (RdRp) |
|  | PHSV |  | PHSV | NC_007749 | Seg-2 (T2) |
|  | PHSV |  | PHSV | NC_007754 | Seg-8 (T13) |
| ***St Croix River virus (SCRV)*** | St Croix River virus |  | SCRV | NC_005997 | Seg-1 (RdRp) |
|  | St Croix River virus |  | SCRV | AF133432 | Seg-2 (T2) |
|  | St Croix River virus |  | SCRV | NC_006004 | Seg-8 (T13) |
| ***Umatilla virus*** | Umatilla virus | USA1969/01 | UMAV | HQ842619 | Seg-1 (RdRp) |
|  | Umatilla virus | USA1969/01 | UMAV | HQ842620 | Seg-2 (T2) |
|  | Umatilla virus | USA1969/01 | UMAV | HQ842626 | Seg-8 (T13) |
| ***Wallal virus (WALV)*** | Wallal virus | Ch 12048 | WALV | AF530084**^*^** | Seg-3 (T2) |
| ***Warrego virus (WARV)*** | Warrego virus | Ch 9935 | WARV | AF530083**^*^** | Seg-3 (T2) |
|  | Warrego virus | V5080 | V5080 | EF213555**^*^** | Seg-3 (T2) |
| ***Wongorr virus (WGRV)*** | Wongorr virus | V195 | WGRV195 | U56990**^*^** | Seg-3 (T2) |
|  | Wongorr virus | V199 | WGRV199 | U56991**^*^** | Seg-3 (T2) |
|  | Wongorr virus | MRM13443 | MRM13443 | U56992**^*^** | Seg-3 (T2) |
|  | Wongorr virus | Paroo River virus | PARV | U56993**^*^** | Seg-3 (T2) |
|  | Wongorr virus | Picola virus | PIAV | U56994**^*^** | Seg-3 (T2) |
| ***Yunnan Orbivirus (YUOV)*** | YUOV | YOV-77-2 | YUOV | AY701509 | Seg-1 (RdRp) |
|  | YUOV | YOV-77-2 | YUOV | NC_007657 | Seg-2 (T2) |
|  | Middle Point Orbivirus | DPP4440 | MPOV | EF591620 | Seg-2 (T2) |
|  | YUOV | YOV-77-2 | YUOV | NC_007663 | Seg-7 (T13) |
| Stretch Lagoon Orbivirus (SLOV) | SLOV | K49460 | SLOV | EU718676 | Seg-1 (RdRp) |
|  | SLOV | K49460 | SLOV | EU718677 | Seg-2 (T2) |

* : Partial sequences only
