# Supplementary material for: Full Genome Sequencing and Genetic Characterization of Eubenangee Viruses Identify Pata Virus as a Distinct Species within the Genus Orbivirus
Source: PLoS One. 2012 Mar 15;7(3):e31911. doi: 10.1371/journal.pone.0031911 (PMC3305294; doi:10.1371/journal.pone.0031911)
Supplement: Table S2 — Percent aa identity of EUBV, TILV and PATAV Polymerase (Pol) protein with other orbiviruses. (DOCX) [file pone.0031911.s003.docx]

**Supplementary data**

**Table S2**: Percent aa identity of EUBV, TILV and PATAV Polymerase (Pol) protein with other orbiviruses

|  | **EUBV** | **TILV** | **PATA** | **Vector** |
| --- | --- | --- | --- | --- |
| EUBV/AUS1963/01 | 100.00 | 92.12 | 64.98 | Culicoides |
| TILV/AUS1978/03 | 92.12 | 100.00 | 64.67 |  |
| PATA/CAF1968/01 | 64.98 | 64.67 | 100.00 |  |
| BTV6w/GQ506536 | 63.41 | 63.18 | 70.25 |  |
| BTV12e/GU390658 | 63.49 | 63.11 | 70.25 |  |
| TOV/GQ982522 | 63.49 | 62.57 | 68.72 |  |
| BTV26/ JN255156 | 63.49 | 62.87 | 69.33 |  |
| EHDV2e/AM744987 | 64.26 | 63.87 | 72.10 |  |
| EHDV1w/AM744977 | 63.26 | 63.64 | 72.41 |  |
| AHSV1/FJ183364 | 58.48 | 58.86 | 59.35 |  |
| EEV/FJ183384 | 52.03 | 52.49 | 54.43 |  |
| CHUV/NC_005990 | 57.39 | 57.62 | 57.64 |  |
| YUOV/AY701509 | 45.88 | 45.42 | 45.83 | Mosquitoes |
| SLOV/EU718676 | 45.61 | 46.08 | 48.95 |  |
| UMAV/ HQ842619 | 46.54 | 46.70 | 49.88 |  |
| PHSV/DQ248057 | 47.18 | 46.80 | 46.90 |  |
| GIV/HM543465 | 47.26 | 47.26 | 47.29 | Ticks |
| KEMV/HM543481 | 47.18 | 47.18 | 47.05 |  |
| LIPV/HM543475 | 46.51 | 46.12 | 46.61 |  |
| TRBV/HM543478 | 46.12 | 46.12 | 46.38 |  |
| SCRV/NC_005997 | 36.08 | 36.63 | 36.90 | Tick (hosts) |
