# Supplementary material for: Full Genome Sequencing and Genetic Characterization of Eubenangee Viruses Identify Pata Virus as a Distinct Species within the Genus Orbivirus
Source: PLoS One. 2012 Mar 15;7(3):e31911. doi: 10.1371/journal.pone.0031911 (PMC3305294; doi:10.1371/journal.pone.0031911)
Supplement: Table S3 — Identity levels in the outer-core protein VP7(T13) of EUBV, TILV and PATAV compared to other Orbiviruses. (DOCX) [file pone.0031911.s004.docx]

**Supplementary data**

**Table S3**: Identity levels in the outer-core protein VP7(T13) of EUBV, TILV and PATAV compared to other Orbiviruses

|  | **EUBV** | **TILV** | **PATA** | **Vectors** |
| --- | --- | --- | --- | --- |
| EUBV/AUS1963/01 | 100.00 | 95.14 | 57.47 | Culicoides |
| TILV/AUS1978/03 | 95.14 | 100.00 | 57.18 |  |
| PATA/CAF1968/01 | 57.47 | 57.18 | 100.00 |  |
| BTV15e/L11723 | 51.58 | 51.29 | 64.37 |  |
| BTV16w/GQ506542 | 52.72 | 53.01 | 65.23 |  |
| TOV/EU839843 | 53.58 | 53.30 | 64.37 |  |
| BTV26/ HM590644 | 54.44 | 54.15 | 64.94 |  |
| EHDV2w/AM745003 | 54.44 | 54.73 | 67.53 |  |
| EHDV2e/AM744993 | 53.87 | 54.15 | 66.95 |  |
| EEV/FJ183391 | 39.14 | 40.29 | 45.11 |  |
| AHSV/HM035361 | 45.27 | 45.56 | 47.55 |  |
| CHUV/NC005988 | 38.79 | 37.93 | 42.49 |  |
| YUOV/NC007663 | 21.49 | 20.63 | 20.75 | Mosquitoes |
| PHSV/NC007754 | 20.69 | 20.98 | 22.25 |  |
| UMAV/ HQ842626 | 26.01 | 25.72 | 25.87 |  |
| BRDV/M87876 | 22.83 | 22.25 | 21.51 | Ticks |
| GIV/ HM543471 | 24.50 | 24.21 | 23.48 |  |
| SCRV/NC006004 | 22.65 | 21.76 | 20.41 | Tick (host) |
