# Supplementary material for: Full Genome Sequencing and Genetic Characterization of Eubenangee Viruses Identify Pata Virus as a Distinct Species within the Genus Orbivirus
Source: PLoS One. 2012 Mar 15;7(3):e31911. doi: 10.1371/journal.pone.0031911 (PMC3305294; doi:10.1371/journal.pone.0031911)
Supplement: Table S4 — Nuclear localization signals (NLS) in NS4 of Eubenangee, Tilligerry and Pata viruses. (DOCX) [file pone.0031911.s005.docx]

**Supplementary data**

**Table S4**: Nuclear localization signals (NLS) in NS4 of Eubenangee, Tilligerry and Pata viruses.

| **Virus** | **Type of signal** | **NLS Position** | **Nucleolar localisation signal (NLS) sequence** | **cNLS score** | **Signal Interpretation** |
| --- | --- | --- | --- | --- | --- |
| **EUBV** | Monopartite | 22-35 | VARPLKRQKTEWGW | 12.5 | Nuclear |
|  | Bipartite | 1-30 | MMKRKAESELKMEIPGLPGVEVARPLKRQK | 6.3 | Nuclear/Cytoplasm |
| **TILV** | Monopartite | 22-35 | VARPRKRQKTELGW | 16 | Nuclear |
|  | Bipartite | 2-29 | KRRAESELTMEIPELPGVEVARPRKRQK | 7.2 | Partially nuclear |
|  | Bipartite | 23-53 | RPRKRQKTELGWKAAVNRAEQSTDQERMEVW | 5 | Nuclear/Cytoplasm |
| **PATAV** | Bipartite | 13-48 | RAMRQRVKKLQFEMWMDSYLLRWDLEEAAEKLAKT | 7.7 | Partially nuclear |
